# HARNESSING THE POWER OF NETWORKS TO ADDRESS SOCIAL ISOLATION, LONELINESS, DEPRESSION, AND ELDER ABUSE

**DOI:** 10.1093/geroni/igz038.1966

**Published:** 2019-11-08

**Authors:** Tobi A Abramson, Berman Jacquelin, Jo Anne Sirey

**Affiliations:** 1 New York City Department for the Aging, New York, New York, United States; 2 Weill Cornell Medicine, White Plains, New York, United States

## Abstract

Challenges to aging and risks to physical health and mortality arise when older adults have untreated mental health needs, experiences social isolation, loneliness, or is a victim of elder abuse. This symposium presents three innovative models that harness the power of networks and collaboration between aging services and an academic medical center to meet the needs of ethnically diverse community seniors. These model programs include: embedded mental health services on-site in community senior centers; Friendly Visiting to homebound seniors; and PROTECT intervention to treat elder abuse victims’ mental health needs. Research from these innovative, collaborative programs indicate that over 50% of senior center members screened positive for depression and anxiety (higher than the national average of 3-20%), social isolation, loneliness, and elder abuse. Of the 75% who engage in treatment, 37.3% and 41% showed a 3-month improvement of depression and anxiety, respectively. For seniors who have a friendly visitor, one-third also suffer from depression and/or anxiety. Three months after being visited by a friendly visitor, 42% and 53% see improvement in loneliness and social isolation, respectively. Among victims of elder mistreatment, 33% screened positive for depression or anxiety and 16% reported suicidal ideation. Clients receiving the PROTECT intervention had a greater decrease in depression, felt services were more useful, and reported greater improvement in the abuse. To find and build strength in age, it is essential that programs and policy be developed to support collaboration and provide the opportunities for building and utilizing networks across different domains of aging.

